# Magnesium and boron isotope evidence for the generation of arc magma through serpentinite-mélange melting

**DOI:** 10.1093/nsr/nwae363

**Published:** 2024-10-17

**Authors:** Xin-Yue Qiao, Jia-Wei Xiong, Yi-Xiang Chen, Jan C M De Hoog, Julian Pearce, Fang Huang, Zi-Fu Zhao, Kun Chen

**Affiliations:** State Key Laboratory of Lithospheric and Environmental Co-evolution, University of Science and Technology of China, Hefei 230026, China; School of Earth and Space Sciences, University of Science and Technology of China, Hefei 230026, China; School of Earth and Space Sciences, University of Science and Technology of China, Hefei 230026, China; State Key Laboratory of Lithospheric and Environmental Co-evolution, University of Science and Technology of China, Hefei 230026, China; School of Earth and Space Sciences, University of Science and Technology of China, Hefei 230026, China; Deep Space Exploration Laboratory, Hefei 230026, China; School of GeoSciences, Grant Institute, University of Edinburgh, Edinburgh EH9 3FE, UK; School of Earth and Ocean Sciences, Cardiff University, Cardiff CF10 3AT, UK; State Key Laboratory of Lithospheric and Environmental Co-evolution, University of Science and Technology of China, Hefei 230026, China; School of Earth and Space Sciences, University of Science and Technology of China, Hefei 230026, China; Deep Space Exploration Laboratory, Hefei 230026, China; State Key Laboratory of Lithospheric and Environmental Co-evolution, University of Science and Technology of China, Hefei 230026, China; School of Earth and Space Sciences, University of Science and Technology of China, Hefei 230026, China; State Key Laboratory of Lithospheric and Environmental Co-evolution, University of Science and Technology of China, Hefei 230026, China; School of Earth and Space Sciences, University of Science and Technology of China, Hefei 230026, China

**Keywords:** arc magma, Mg isotope, B isotope, serpentinite, crust–mantle interaction

## Abstract

Serpentinites play a crucial role in mass transport and volatile recycling in subduction zones, yet the mechanism for their contribution to the formation of arc magma remains elusive. Here, we investigate this issue by examining the magnesium (Mg) and boron (B) isotope compositions of volcanic rocks and forearc serpentinites from the South Sandwich Island arc. The volcanic rocks display δ^26^Mg values ranging from −0.25‰ to −0.06‰ and δ^11^B values ranging from +9.6‰ to +16.5‰, while the forearc serpentinites exhibit δ^26^Mg values of −0.21‰ to −0.02‰ and δ^11^B values of +5.2‰ to +9.8‰. Given the substantial contrast in both Mg and B contents between mantle rocks and fluids, the combined heavy Mg–B isotope compositions of volcanic rocks pose a challenge to traditional arc formation models, i.e. flux melting of depleted subarc mantle metasomatized by slab-derived fluids. Although an alternative model involving flux melting of dehydrated serpentinites can partly account for the heavy Mg isotope compositions of arc magmas, it is difficult to simultaneously explain the B isotope and trace-element compositions. Instead, these distinct compositions can be adequately explained by partial melting of a serpentinite-dominated mélange beneath the volcanic arc. Given that arc magmas exhibiting coupled heavy Mg–B isotope compositions are increasingly reported, we propose that serpentinite-mélange melting represents an effective and geochemically self-consistent mechanism for transferring signatures of subducted slabs to the overlying mantle source. This process can be significant in subduction zones with prominent forearc mantle erosion or those involving considerable amounts of slab-hosted serpentinite.

## Teaser

This study reports the first set of combined heavy Mg and B isotope compositions of arc magmas and associated forearc serpentinites from the South Sandwich island arc, and proposes that such compositions can be formed by the diapiric rise and melting of forearc serpentinite dominated mélanges.

## INTRODUCTION

The formation of arc magmas has been traditionally attributed to the partial melting of mantle wedge peridotite—a process driven by fluids derived from the subducting slab [1,2]. Numerous studies have focused on distinguishing the contributions of various subducted components, primarily composed of sediment, altered oceanic crust (AOC) and serpentinite, to the mantle source of arc magmas. Serpentinite, known for its capacity to accommodate water and boron (B) [3], is characterized by heavy B isotope compositions that are distinct from those of sediment and AOC at subarc depths [4–6]. Combined with the fluid-mobile behavior of B during serpentinite dehydration [7,8], B isotopes serve as a powerful tool for tracing the contribution of serpentinite to the source of arc magmas in subduction zones [9–12].

Island arc volcanic rocks typically have higher B contents and δ^11^B values than mid-oceanic ridge basalts (MORB) [5]. This has been traditionally attributed to their being sourced from enriched mantle hybridized by fluids derived from subducted AOC and/or sediments [2,13]. More recently, growing evidence indicates that serpentinite plays an important role in the generation of arc lavas—particularly those with high δ^11^B values (>+5‰) [4,5,9–12,14]. However, the exact mechanism and process for the contributions of serpentinites are still unclear. In addition to the well-established flux melting model, diapirism within the mantle wedge, potentially as part of a mélange, may also play an important role [15–17]. Moreover, across-arc geochemical variability in volcanic arc magma could provide insights into the composition of mantle wedges and slab dehydration or melting processes [9,10,18]. The correlations of various isotope tracers (such as B–Sr–Nd isotopes) and trace-element ratios (such as B/Nb) along the arc can also effectively reflect the influence of serpentinite components in subduction zones [9,10].

Arc magmas with δ^26^Mg values that are higher than those of MORB have been increasingly reported [19–22]. The observed heavy Mg isotope data were attributed to either fractional crystallization and/or crustal assimilation processes or a mantle source that has been metasomatized by slab components [20,23,24]. Regarding fractional crystallization, a significant increase in the δ^26^Mg value has been predominantly observed in differentiated magmatic rocks with MgO < ∼5 wt% [19–24]. Whether this process can account for the Mg isotopic fractionation in more primitive arc magmas remains uncertain, highlighting the necessity for examining the Mg isotope systematics of high-Mg, relatively unfractionated rocks. Another explanation for the heavy Mg isotope compositions in arc magmas is that they primarily reflect a metasomatized mantle source by slab-derived fluids [20]. However, due to the substantial difference in Mg contents between mantle rocks and aqueous fluids, the mass proportion of infiltrating fluids would need to be exceptionally high (>50%) [25]. This high fluid proportion clearly contradicts the constraints provided by B isotope systematics, which suggest a fluid contribution of <5% [11,12]. This apparent paradox indicates that the mechanism for mantle source modulation by subducted components may be more complex than a simple fluid-flux melting process. Other mechanisms, such as the melting of dehydrated forearc serpentinite or the involvement of serpentinite-bearing mélange diapirs, should be considered [9,16,17].

The South Sandwich Island (SSI) arc in the South Atlantic is an intra-oceanic arc characterized by a young age of <3 Ma, a simple tectonic setting and a considerable distance away from any continental crust [26]. SSI arc lavas span large MgO contents and have the highest δ^11^B values among worldwide arc magmatic rocks, which has been attributed to fluids derived from forearc serpentinites that were eroded and transported to subarc depths [11]. These unique samples provide an excellent opportunity to investigate the contribution of serpentinites to the mantle and to study the mechanism of crust–mantle interactions in subduction zones.

In this study, we present the first set of combined Mg and B isotope compositions of arc magmas and associated forearc serpentinites from the SSI arc. These arc magmas simultaneously exhibit high δ^11^B and δ^26^Mg values, which are difficult to explain solely by fluid-flux melting or magmatic evolution. We propose that the diapiric rise and partial melting of mélanges composed of forearc serpentinite and minor sediments can account for the geochemical compositions of SSI arc magmas. Given the increasing reports of coupled heavy Mg–B isotope compositions in both serpentinites and arc rocks, we argue that, in addition to the traditional flux melting model, the diapirism of serpentinite-bearing mélanges may also play a significant role in the generation of arc magmas.

## RESULTS

**Figure 1.**
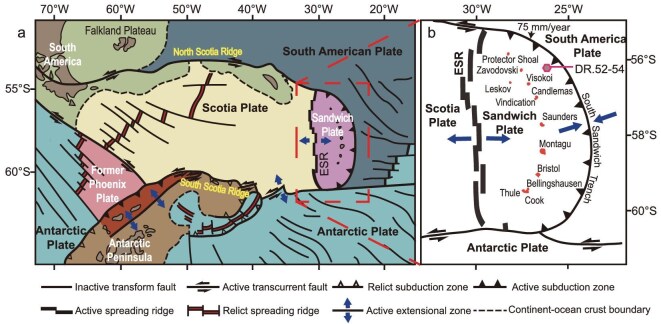
(a) Tectonic setting of the South Sandwich Island (SSI) arc–East Scotia Ridge (ESR) region (modified after [28]). CSS, Central Scotia Sea; ESS, East Scotia Sea; WSR, West Scotia Ridge; WSS, West Scotia Sea. (b) Detailed location of the SSI arc (modified after [26]). The volcanic samples are from islands on the Sandwich Plate. The hexagon indicates the location of the dredged serpentinized peridotites [27].

We analysed the boron and magnesium isotope compositions of volcanic samples and associated forearc serpentinites from the SSI arc-basin system. The general petrology and geochemistry have been reported previously [26,27]. The arc lavas were collected from 11 main islands on the Sandwich microplate (Fig. 1), most of which belong to the (low-K) tholeiitic magma series, but some lavas are calc-alkaline [26]. The extensively serpentinized forearc peridotites were recovered from the inner wall of the South Sandwich Trench during the dredging program undertaken by dredges 52–54 from the British Antarctic Survey [26,27,29] (Fig. 1). The slab depth beneath the SSI arc volcanoes ranges from 80 to 155 km based on the data from Hayes *et al.* [30] (Fig. S1).

**Figure 2.**
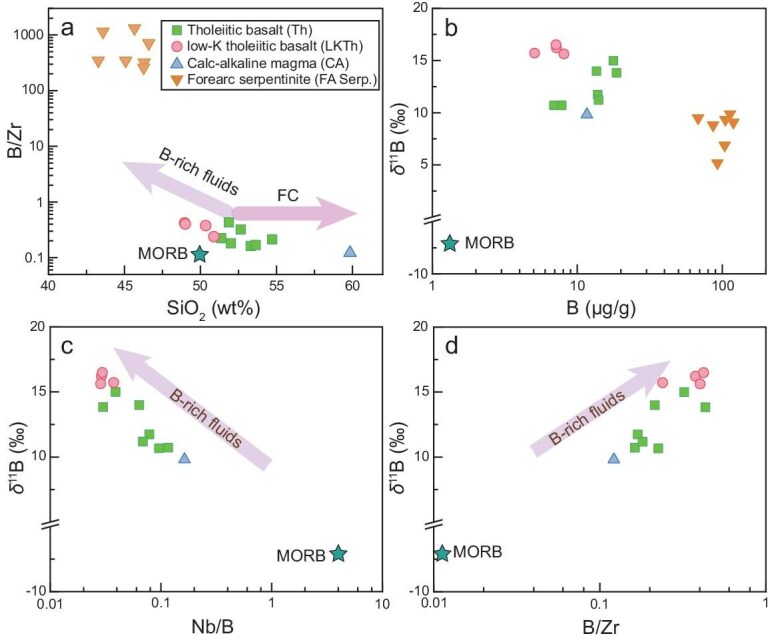
Boron element and isotope variations in arc magma and forearc serpentinite from the SSI. (a) B/Zr vs. SiO_2_. δ^11^B vs. (b) B concentration, (c) Nb/B ratio and (d) B/Zr ratio of arc magma and forearc serpentinite. The error of the δ^11^B is smaller than the symbol. Both the major and trace-element data are from [26,27] and the MORB data are from [31,32].

Boron contents range from 5.1 to 18.6 μg/g for SSI arc magmas and from 59 to 119 μg/g for the serpentinites (Fig. 2) (Table S1). The SSI arc magmas have the highest δ^11^B values worldwide, ranging from +9.6‰ to +16.5‰, while the forearc serpentinites have δ^11^B values ranging from +5.1‰ to +9.8‰. Both arc rocks and forearc serpentinites exhibit δ^11^B values that are systematically higher than that of the mantle of approximately −7‰ [31].

**Figure 3.**
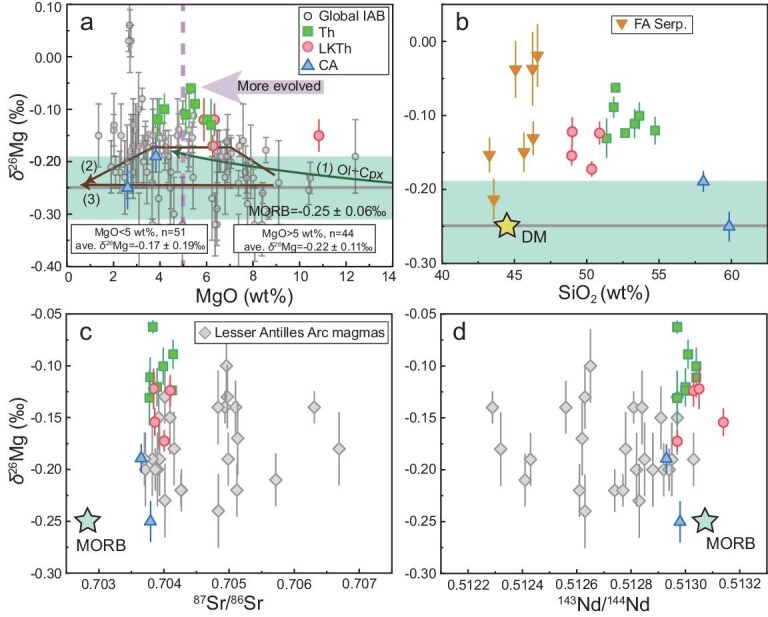
Plots of δ^26^Mg vs. (a) MgO, (b) SiO_2_, (c) ^87^Sr/^86^Sr ratios and (d) ^143^Nd/^144^Nd ratios of arc magma and forearc serpentinite from the SSI. Data for Lesser Antilles Arc (LAA) magmas and mantle from [20] are shown for comparison. Mg isotope data for global island arc magmas are from [19–21,23]. The line (1) represents the modeled δ^26^Mg evolution during the co-crystallization of 20% olivine and 20% clinopyroxene [21]. The lines (2) and (3) represent the reported Mg isotope evolution trend of MORB from East Pacific Rise and Kilauea Iki lava, respectively [31,34]. The isotopic compositions of MORB are from [32,33].

In terms of Mg isotopes, the arc rocks have δ^26^Mg values ranging from −0.25‰ to −0.06‰ (Table S2). Among the subgroups, the low-K tholeiites and normal tholeiites exhibit δ^26^Mg values of −0.17‰ to −0.12‰ and −0.13‰ to −0.06‰, respectively, which are significantly greater than the MORB of −0.25‰ ± 0.06‰ [33]. The calc-alkaline samples with the lowest MgO contents (2.6–3.8 wt%) display relatively low δ^26^Mg values of −0.25‰ to −0.19‰ (Fig. 3). The forearc serpentinites show a δ^26^Mg range of −0.21‰ to −0.02‰, broadly overlapping the arc magmas. There is no correlation between the Mg isotope compositions and geographical location or slab depths, while the B/Nb ratios, δ^11^B values and ^87^Sr/^86^Sr ratios tend to decrease with increasing slab depths (Fig. S2).

## DISCUSSION

### The origin of heavy B–Mg isotope compositions in SSI arc magmas

The processes of subduction material recycling in the SSI arc are examined in the context of B–Mg isotope systematics. Boron contents in the arc magmas range from 5.1 to 18.6 μg/g, which are significantly higher than the MORB value of ∼1.3 μg/g [31]. The ratio of B to fluid immobile elements such as Zr or Nb in arc rocks can be used to infer the nature of the mantle source [11,12,31,35]. Notably, while B and Zr do not fractionate from each other during partial melting and magma differentiation, Nb is more incompatible than B and Zr [31]. However, generally consistent trends between B/Zr and B/Nb (Fig. 2) suggest that the difference in compatibility between Nb and B does not significantly affect the evaluation. In the SiO_2_ vs. B/Zr plot of the SSI arc rocks (Fig. 2a), fractional crystallization leads to SiO_2_ enrichment while maintaining a constant B/Zr ratio, confirming the similar partitioning of both elements. The enrichment of B in both arc rocks and forearc serpentinites suggests the incorporation of B-rich fluids (Fig. 2b). Specifically, as fractional crystallization has a negligible effect on the δ^11^B values of residual melt [14,36], the correlation between B/Zr (and Nb/B) ratios and δ^11^B values clearly shows the involvement of serpentinite or serpentinite-derived materials in the source of the arc magmas (Fig. 2c and d). Indeed, the very high δ^11^B values of SSI arc rocks have been interpreted to result from a hybridized mantle infiltrated by fluids from forearc serpentinites, which were transferred to subarc depths via subduction erosion [11].

The high δ^26^Mg values observed in both SSI arc magmas (−0.25‰ to −0.06‰) and associated forearc serpentinites (−0.21‰ to −0.02‰) are notably higher than those of MORB (Fig. 3), which, again suggests a potential contribution of the forearc serpentinite component to the formation of SSI arc rocks. Given that the large range of δ^26^Mg values of these arc rocks do no correlate with either large ion lithophile elements or Sr–Nd isotope compositions (Fig. 3), it can be inferred that crustal assimilation does not account for the elevated δ^26^Mg values in the magmas. It is thus crucial to clarify whether these heavy Mg isotopic compositions in arc magmas represent a mantle source signal or are instead the result of magmatic differentiation processes. While mantle melting has a negligible effect on Mg isotope fractionation [37,38], previous studies have documented the influence of fractional crystallization of basaltic melts [21,24,39,40]. A negative Mg isotope fractionation factor between olivine and melt was identified, suggesting that the separation of olivine could lead to the enrichment of heavy Mg isotopes in evolved residues [21]. The modeling results of δ^26^Mg evolution during the co-crystallization of 20% olivine and 20% clinopyroxene shows that the magmatic variation in less-evolved arc magmas does not exceed the MORB range [21] (Fig. 3a). In addition, this crystallization effect may be partially offset by the simultaneous separation of spinel/chromite, which are preferentially enriched in isotopically heavy Mg [38,39]. A compilation of global island arc basalts in Fig. 2a demonstrates that most less-evolved samples with MgO contents of >5 wt% exhibit δ^26^Mg values that are similar to those of MORB within analytical uncertainty, whereas highly evolved samples tend to show elevated δ^26^Mg values. This observation supports previous findings that significant Mg isotope fractionation occurs due to crystal fractionation in highly evolved samples [21,40]. However, the overall increase in the δ^26^Mg value caused by crystal fractionation is typically within 0.07‰ [21,40], which is close to the analytical uncertainty. Therefore, δ^26^Mg values of the arc basalts may slightly increase due to the crystallization of olivine in the initial crystallization stage, but the increase is too limited to show an observable difference compared with those of MORB [33,37] (Fig. 3a). In this regard, the effect of crystal fractionation may be overestimated and requires further constraints.

The SSI arc magmas, characterized by high δ^26^Mg values in conjunction with relatively high MgO contents (up to ∼11 wt%), represent a more primitive magma (Fig. 3a). Their Mg isotope compositions are inconsistent with the evolution trend caused by fractional crystallization [21,40]. Consequently, the heavy Mg isotope compositions of these unique SSI arc magmas provide compelling evidence that crystal fractionation has an insignificant effect on their elevated δ^26^Mg values, which are primarily inherited from the mantle source (δ^26^Mg > −0.15‰ ∼ −0.10‰). The lower δ^26^Mg values observed in the two calc-alkaline samples with low MgO, TiO_2_ and FeO contents (Fig. 3 and Fig. S3) may be attributed to the crystallization of titanomagnetite during the later stage of magma differentiation [40] or to the mantle source with normal Mg isotope compositions.

### Serpentinite-mélange melting to form SSI arc magma

**Figure 4.**
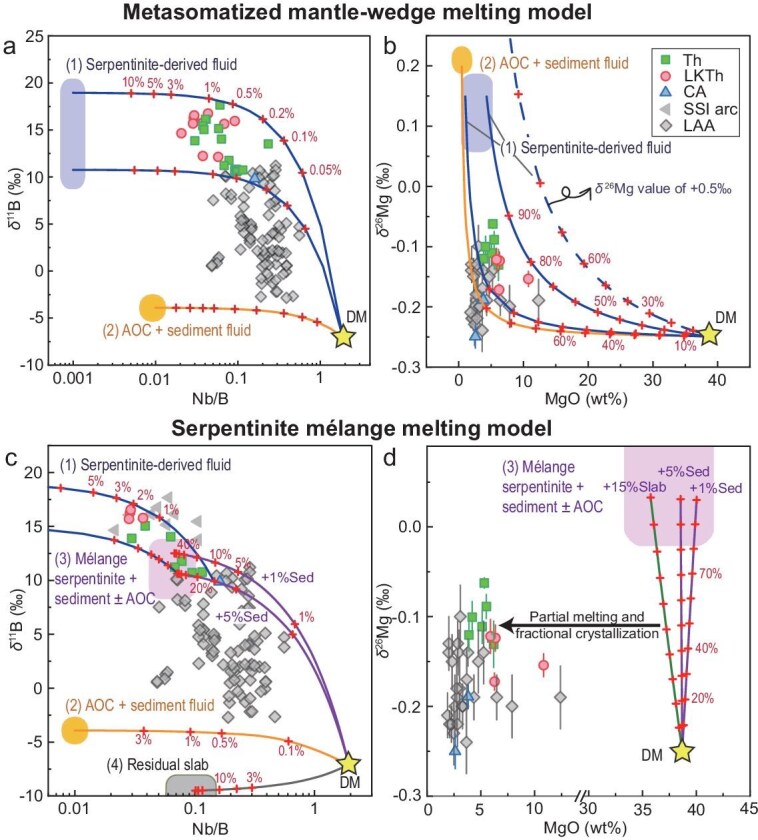
Mg–B isotope modeling for arc magma genesis. (a) and (b) Modeling results showing the contamination of depleted mantle (DM, stars) [31,42] by (1) serpentinite-derived fluids and by (2) fluids derived from the uppermost slab; the dotted line represents in panel (b) serpentinite-derived fluid with high δ^26^Mg of +0.5‰. (c) and (d) Modeling results of mixing DM with (3) a serpentinite-dominated mélange and (4) the subducted slab. Given the lower δ^11^B values of forearc serpentinite than SSI arcs, we also consider the possible contributions of serpentinite-derived fluids at subarc depth. The numbers along each mixing line represent the mass proportions of recycled materials. The error bars on the δ^11^B values are smaller than the symbol size. See ‘Materials and methods’ section for modeling details.

Various processes have been proposed for the contributions of material from the subducted slab to the mantle. A commonly proposed model for arc magma formation is flux melting, which involves the partial melting of a depleted mantle wedge metasomatized by slab-derived aqueous fluids or hydrous melts at subarc depths [1,41]. More recently, diapiric rise and melting of high-pressure mélanges that initially formed at the slab–mantle interface were proposed as another important mechanism for arc magma generation [16,17]. We conducted geochemical mixing modeling based on SSI arc rocks to provide further insights into the dynamics of material recycling and arc magma generation in subduction zones (Fig. 4). The parameters used in the modeling are provided in Table S3 and the details of the model are described in the ‘Materials and methods’ section.

The geochemical characteristics of SSI arc magmas—particularly their coupled heavy Mg and B isotope compositions—cannot be explained by fluid/melt metasomatism of the mantle wedge. This is due to the low B and Mg contents and low δ^11^B values of the fluid/melt generated by metasediments and AOC at subarc depths [5,43] (Fig. 4c). These features also present challenges for the traditional fluid-flux melting model driven by serpentinite-derived fluids as proposed by Tonarini *et al.* [11] and Cooper *et al.* [12]. While such a model can reasonably explain the high δ^11^B values of SSI arc magmas (where fluid addition does not exceed 3% in mass proportion, Fig. 4a), it cannot simultaneously account for the high δ^26^Mg values, given the contrasting Mg contents between mantle rocks and aqueous fluids (Fig. 4b). Although serpentinite-derived fluids are expected to contain relatively high Mg contents compared with those derived from crustal materials [8,25,43], it still requires exceptionally large amounts of fluids to account for the observed high δ^26^Mg values of SSI arc rocks. For instance, even under extreme upper limit estimates of MgO contents (5 wt%) and δ^26^Mg values (+0.5‰) of serpentinite-derived fluids, the required fluid mass proportion still exceeds 50%, which is unreasonable (Fig. 4b). Furthermore, the strikingly contrasting fluid proportions inferred from Mg and B isotopic constraints pose a challenge to this scenario. This discrepancy necessitates an alternative mechanism to explain the observed geochemical signatures in SSI arc magmas.

Serpentinites can occur in various tectonic settings—as abyssal serpentinites on the ocean floor, as forearc serpentinites formed by slab fluid metasomatism and as slab mantle resulting from bending near the trench [9,44]. Due to their low density and viscosity, these serpentinites can be subducted and transported into the hot mantle wedge, where they rise and may subsequently undergo flux melting induced by slab-derived fluids [9,10]. Notably, the dehydration of serpentinite, despite progressive changes in *P–T* conditions, does not significantly modify the heavy Mg isotope composition of the residues due to the limited amount of Mg in the fluids. High-pressure serpentinites and their dehydration products can still display high δ^11^B values, even exceeding +20‰ [4,45,46], while retaining significant B due to its high solubility in secondary olivine [46–48]. Given that the temperature at the slab–mantle interface of subarc depths is too low to induce the melting of refractory serpentinite [49], the scenario involving serpentinite diapirism can be considered as a hybrid fluid-flux melting model. Considering the depleted nature of serpentinites [27,50], the addition of a sediment component is required to account for the high trace-element compositions of arc magmas [26,51,52]. Partial melting modeling based on a composite source composed of forearc serpentinites and depleted mantle, along with Sr–Nd isotope mixing modeling, consistently suggests that the mass proportion of the sediment added to the mantle source is less than ∼3% (see ‘Materials and methods’ for details) (Figs S5 and S6). This observation aligns with previous work showing that the addition of <6% of sediment to the mantle can effectively account for the incompatible element contents of global arc magmas [51].

Furthermore, the sediment component added to the SSI arc mantle source was documented to exhibit similar Nb/La ratios to those of the bulk sediment [52]. This observation can be explained by the sediment being either added in the form of a bulk solid or through sediment melt without changing the Nb/La ratio. The latter can be achieved only when rutile is absent during sediment melting [52]. However, high-*P*/high-*T* experimental results, using starting materials that are similar to those of South Sandwich sediments—especially in terms of Ti and Fe contents—have indicated the presence of rutile at pressures exceeding 2 GPa during sediment melting [53,54]. To further verify this point, we conducted phase equilibrium modeling by using South Sandwich sediment as the bulk composition (see ‘Materials and methods’ for details). The results also confirm the presence of rutile during sediment melting (Fig. S7). Thus, the Th/La, Sm/La and Nb/La systematics of SSI arc magmas suggest that the sediment was recycled in the form of a bulk solid rather than as a melt. This is consistent with previous constraints by using Sr–Nd isotope compositions [17], as well as our modeling results (Fig. S6B). Notably, the addition of sediment in bulk solid form does not favor the hybrid flux melting model, but instead aligns well with the serpentinite-dominated mélange model (Fig. S6).

The Mg–B isotope data can also be reconciled by considering a serpentinite-dominated mélange melting process. This scenario involves the physical mixing of serpentinite and minor sediments to form low-density diapirs. Considering the observed high δ^11^B and δ^26^Mg values in SSI forearc serpentinites (Figs 2b and 3c), and the fact that forearc serpentinites can be transferred to subarc depths through mantle flow or subduction erosion [11,29,35,55], we suggest that SSI forearc serpentinites may have served as the main constituents of the mélange materials. Alongside the widespread high δ^11^B values, both mantle wedge serpentinite and seafloor serpentinites can also show elevated δ^26^Mg values [56–58] (Fig. S4), which are attributed to serpentinization and/or chemical weathering [56,57,59].

**Figure 5.**
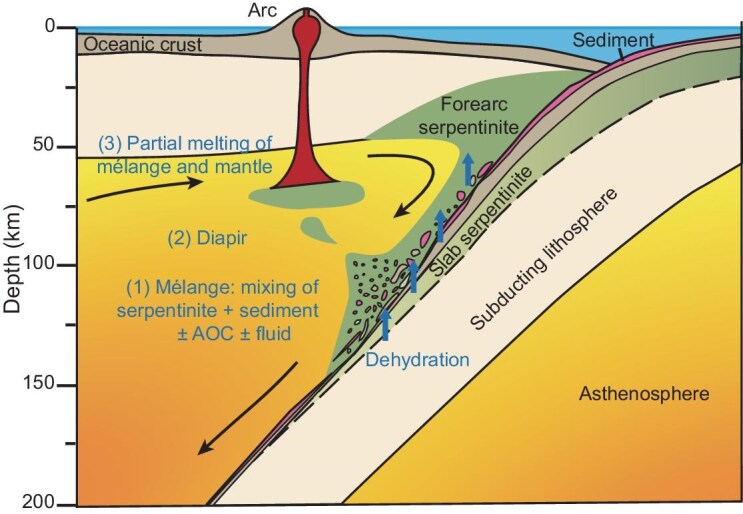
The serpentinite-dominated diapiric mélange melting model for the origin of the SSI arc magmas, modified from [17]. The serpentinite-dominated mélange was initially formed at the slab–mantle interface, which is primarily composed of forearc serpentinite along with minor sediments and possibly other slab-derived materials. The mélange rises as diapirs into the overlying hot mantle wedge due to its buoyancy and low viscosity, which then melts alongside the surrounding mantle rocks and eventually forms SSI arc magmas.

The quantitative modeling for the process described above is shown in Fig. 4, with a schematic illustration provided in Fig. 5. Our results reveal that a composite mantle source constituting 20%–70% (by mass) of the mélange can effectively explain the high δ^26^Mg values of the SSI arc magmas (Fig. 4). However, as the δ^11^B values of SSI forearc serpentinites are lower than those of associated arc magmas, the interpretation of the B isotope systematics requires a more complex scenario (Fig. 4). This feature could be attributed to the fact that the serpentinites dredged from the SSI trench may not fully represent all serpentinites eroded in the forearc region, as global reports have shown that forearc serpentinites can exhibit higher δ^11^B values (can exceed +20‰) [60,61]. Alternatively, the forearc serpentinite-dominated mélange may have undergone additional infiltration by ^11^B-rich fluids, which derived from slab serpentinite that is known to maintain high δ^11^B values even at great depths [4,45,46] (Fig. 4c). In either scenario, the melting of serpentinite-dominated mélange diapirs provides a plausible mechanism for the coupled heavy Mg and B isotope compositions observed in the SSI arc magmas. Furthermore, the interaction between melts generated from the partial melting of serpentinite-dominated mélange and mantle peridotite can yield arc-like major element compositions, further supporting the hypothesis that mélanges can serve as a potential source of arc magmas [62].

Previous studies have documented cross-arc geochemical variations within many island arc regions, including progressive change in the concentrations of fluid-mobile elements and the isotopic compositions of B–Sr–Nd–Pb–Mo systems [2,10,18,63–65]. For example, it has been noted that the contents of fluid-mobile elements, such as B and Pb, decrease with increasing depths of arc magma formation [2,18], which was interpreted as being driven by the dehydration of the subducting slab at varying depths. In addition, an increase in the concentration of elements such as Th and Hf further requires the involvement of a melt component, likely sourced from subducted sediments [66]. However, the scenario involving a mantle source metasomatized by a fluid or melt component makes it difficult to explain the heavy B–Mo isotope compositions observed in some arc magmas [11,12,65,67] because heavy isotopes would typically be depleted during the progressive subduction before subarc depths [5,68]. Recently, a multistage model has been proposed to account for the cross-arc geochemical variations, particularly regarding the B–Mo–Sr–Nd-Pb isotope compositions from the Mariana and Kurile arcs [9,10,64]. According to this model, the heavy B–Mo isotope compositions in arc magmas can be primarily attributed to the dehydration of forearc serpentinites at frontal arc depths, whereas the lighter B–Mo isotope compositions in rear arc rocks were due to the distillation of B and Mo from sediments or AOC by fluids derived from serpentinite.

The SSI arc magmas display similar cross-arc geochemical variations, with the frontal arcs showing higher B/Nb ratios, δ^11^B values and radiogenic Sr isotope compositions than the rear arcs (Fig. S2). In our proposed scenario, forearc serpentinites were scraped off and mixed with minor amounts of sediments and AOC, forming a serpentinite-dominated mélange. The diapiric rise of this buoyant mélange into the wedge can explain the heavy Mg–B isotope compositions of the SSI arcs. The distance-related geochemical variations can be accommodated by the decreasing amounts of crustal-derived materials or the preferential releasing of fluid-mobile elements of mélanges at shallower depths. Consequently, frontal arc rocks exhibit higher B/Nb ratios, heavier B isotope compositions and more radiogenic Sr isotope compositions relative to rear arc rocks. Notably, the suggested model emphasizes the role of serpentinite over previously proposed sediment-dominated mélange model [16], in view of the coupled heavy Mg–B isotope compositions of magmatic products. In addition, the serpentinite-dominated mélange diapir in the wedge mantle may undergo additional metasomatism by fluids or melts derived from the descending slab, thereby enhancing the crustal-derived signals in arc magmas.

### Implication for global arc magma formation

Recently, there has been increasing emphasis on the significant role of serpentinite in geochemical cycling and arc magma generation, highlighting the importance of Mg–B–Mo isotope systematics in discerning serpentinite signatures in subduction zones [6,9,10,12,25,69,70]. Although combined Mg–B isotope studies of arc magmatism are still rare, coupled heavy isotope signatures are increasingly reported [5,20,21,71,72]. For example, Du *et al.* [71] reported arc rocks in Eastern Tianshan, China, with coupled heavy Mg and B isotope compositions (δ^26^Mg = −0.23‰ to −0.13‰, δ^11^B = −0.04‰ to +1.08‰), although they ascribed these signatures to the contribution of serpentinite-derived fluids. Similar isotopic signatures were found in magmas from the Lesser Antilles Arc (LAA), where magmas from the central islands show high δ^26^Mg values of −0.25‰ to −0.10‰ and high δ^11^B values of +2.3‰ to +11.2‰ [12,20]. As discussed earlier, a fluid-mediated metasomatic process would require an exceptionally high fluid mass proportion, which contradicts the constraints imposed by B isotopes (Fig. 4a and b). Trace-element and Sr–Nd–Pb isotope data suggest that the LAA arc lavas contain considerable crustal components in the mantle source [20]. In the context of a mélange model, minor additions of sediments do not significantly alter the Mg–B isotope compositions but can notably shift the Nb/B ratio of the mantle (Fig. 4c). Our model, which involves the mixing of mélange melt, slab-derived fluids and depleted mantle rocks, can also be applied to explain the Mg–B isotope systematics of LAA magmas (Fig. 4c and d). This integrated approach provides a comprehensive framework for understanding the geochemical signatures of arc magmas.

As such, our proposed serpentinite-dominated mélange melting model can effectively account for the formation of arc magmas exhibiting coupled heavy B and Mg isotope signatures. These signatures are increasingly observed in arc magmas [5,20,21,71,72]. In subduction zones where forearc erosion is significant (such as the SSI arc and Mariana arc) [29,73], diapiric rise and partial melting of a mélange predominantly composed of forearc serpentinite may be applicable for the formation of magmas in these settings. Seafloor serpentinites commonly occur at ultraslow to slow spreading ridges [74,75], such as the South American–Antarctic ridge that is associated with the SSI arc system [76]. Additionally, serpentinization also occurs in plate-bending regions where the slab enters subduction zones [75,77]. As serpentinization and/or seafloor weathering of abyssal peridotites can lead to heavy Mg–B isotope compositions in serpentinites [56,59,76], the incorporation of such serpentinites into the mélange can also be considered as an important reservoir for the generation of coupled heavy Mg–B isotopes in arc magmas. The compositions of arc magmas from different localities are influenced by many factors, including the nature of the mantle source, the thermal structure of subduction zones, the degree of partial melting and magmatic differentiation, which are ultimately dictated by the compositions of metasomatic agents in a fluid-flux melting process or material constituents in a diapiric mélange melting scenario. Given the complexity of geochemical compositions observed among different arcs, variable constituents of the mélange may be needed. Our study on SSI arc magmas suggests the important role of serpentinite-dominated mélanges in the formation of global arc magmas with coupled heavy Mg–B isotope compositions (Fig. 5).

## MATERIALS AND METHODS

### Boron content and B isotope analysis

The whole-rock B elemental and isotopic analyses were performed at the State Key Laboratory of Isotope Geochemistry, Guangzhou Institute of Geochemistry, Chinese Academy of Sciences. The sample preparation followed the methods described by Wei *et al.* [78]. Briefly, ∼150 mg of powder was weighed into a polypropylene centrifuge tube and fully digested with HF–H_2_O_2_–mannitol at 60°C for 1 week. Subsequently, the sample solutions were diluted with Milli-Q water and separated using AG MP-1 anion-exchange resin. The resulting solution was further diluted for B content and isotope measurements. The B concentration was determined by using ICP–AES (Inductively Coupled Plasma-Atomic Emission Spectrometry) and the analytical uncertainty was generally less than ±5%. The B isotopic composition was analysed by using a Neptune Plus MC–ICP‒MS and the results are reported as δ^11^B (δ^11^B = 1000 × [(^11^B/^10^B)_Sample_/(^11^B/^10^B)_NBS951_ − 1]). The B contents and δ^11^B values for the external reference materials are consistent with the recommended values within error, validating the reliability of the data (Table S1).

### Mg–Sr–Nd isotope analysis

Mg isotopes were obtained by following the method of An *et al.* [79] at the CAS Key Laboratory of Crust-Mantle Materials and Environments, University of Science and Technology of China (USTC), Hefei, China. Appropriate amounts of whole-rock powders were fully digested with a mixture of concentrated HF-HNO_3_ to obtain ∼20 μg of Mg for chemical purification. Mg purification involved two cycles of chromatography using Bio-Rad AG50 W-X12 resin columns. Mg isotope analysis was performed by using a Thermo-Scientific Neptune Plus MC–ICPMS. The mass bias of the instrument was calibrated by using the sample-standard bracketing method with DSM-3 as the standard. The results are reported in delta notation relative to DSM-3: δ*^x^*Mg = [(*^x^*Mg/^24^Mg)_Sample_/(*^x^*Mg/^24^Mg)_DSM-3_ – 1] × 1000, where *x* = 25 or 26. The long-term external precision for δ^26^Mg values is better than ±0.05‰ [79]. During the analytical session, the δ^26^Mg values of the USGS (United States Geological Survey) reference materials BCR-2, BHVO-2 and AGV-2 were identical within error with established values [37,79] (Table S2). The duplicate samples processed via the same procedure also gave identical δ^26^Mg values within error (Table S2). The plot of δ^25^Mg vs. δ^26^Mg from our data showed a linear trend with a slope of ∼0.520 (Fig. S8), consistently with the theoretical mass-dependent fractionation values [80].

Whole-rock Sr–Nd isotope compositions were measured at the USTC by following the chemical separation and analytical protocol described by Ma *et al.* [81]. In brief, ∼100 mg of sample powder was completely digested by using a mixture of HF–HNO_3_–HCl in capped beakers at 120°C for 1 week. Sr–Nd separation and purification were achieved by using cation exchange chromatography and Sr was further purified by using a Sr-specific resin. The purified Sr–Nd solutions were measured by using a Thermo-Scientific Neptune Plus MC‒ICP‒MS. The isotopic mass fractionations of Sr and Nd were corrected by normalizing ^86^Sr/^88^Sr to 0.1194 and ^146^Nd/^144^Nd to 0.7219, respectively. Multiple international standards, including NBS987 for Sr and JNdi Nd for Nd, were measured for quality control. The USGS reference materials BHVO-2 and BCR-2 were consistent with previously reported values within error [81] (Table S2).

### Mixing models for arc formation

The detailed compositions used in the modeling are presented in Table S3. For depleted MORB mantle (DMM), the parameters are set as follows: B concentration of 0.077 μg/g with a δ^11^B value of −7.1‰ [31], Nb concentration of 0.148 μg/g, MgO concentration of 38.7 wt% [36] and δ^26^Mg value of −0.25‰ [32]. Considering the relatively high B contents of SSI forearc serpentinites, the initial B concentration is assumed to be 60 μg/g [11,60], which experienced a significant loss of B during dehydration via subduction to subarc depths [5,15,82]. Therefore, the final meta-serpentinite is estimated to have a B content of ∼15 μg/g and a δ^11^B value of +13‰, with a Nb/B ratio of 0.0007 according to the distribution coefficient of Nb from Kessel *et al.* [83]. Similarly to previous models, the ranges of B concentrations and δ^11^B values of serpentinite-derived fluids are set as from 325 μg/g and +19‰ to 289 μg/g and +11‰, respectively, with a Nb/B ratio of 0.001 [4,11,12]. The effect of different incompatibilities between Nb and B [31] on the overall discussion is minimal, which will slightly increase the mass proportion of fluids (less than ∼1%).

The MgO contents for SSI forearc serpentinites are from Pearce *et al.* [27] and dehydration does not significantly modify their MgO contents. As serpentinites with high δ^26^Mg values are globally observed [19,57–59], the initial δ^26^Mg value of mélange serpentinite is assumed to be ∼40‰. The MgO contents of serpentinite-derived fluids are assumed to range from 1.0 to 4.3 wt% based on fluid inclusion results from high-pressure metaperidotites [8]. Although serpentinite-derived fluids are suggested to have relatively high δ^26^Mg values, the exact fractionation factor during dehydration remains poorly constrained. We assume the high δ^26^Mg values of serpentinite-derived fluids (+0.15‰ to +0.50‰) to demonstrate that the addition of fluids to the mantle cannot effectively affect the Mg isotope compositions of Mg-rich mantle.

The initial compositions of the subducted sediments and AOC followed those of previous studies [11,13,84–86] and are listed in Table S3. The uppermost slab is assumed to be composed of 90% AOC and 10% sediment in mass proportion [11]. It is assumed that the progressive subduction of sediments will result in the loss of >60% of the initial B content [5,87]. The MgO content of the slab-derived fluids is estimated as a maximal value based on the mineral-fluid partition coefficient of Mg [88]. Since there are no convincing data for the Mg isotope compositions of slab crust-derived fluids, we set an extremely high δ^26^Mg value of +0.20‰ to illustrate the limited effect of slab crust-derived fluids [89]. Additionally, it is expected that the dehydration process would not significantly change the Mg isotopic composition of the residual slab [58] due to the low Mg content of the fluids [90].

## Supplementary Material

nwae363_Supplemental_Files
