# Supplementary material for: Magnesium and boron isotope evidence for the generation of arc magma through serpentinite-mélange melting
Source: Natl Sci Rev. 2024 Oct 17;12(1):nwae363. doi: 10.1093/nsr/nwae363 (PMC12089755; doi:10.1093/nsr/nwae363)
Supplement: nwae363_Supplemental_Files [file nwae363_supplemental_files.zip › NSR_MS-2024-1041 Supporting Information.docx]

**Supporting Information for**

Magnesium and boron isotope evidence for the generation of arc magma through serpentinite mélange melting

Xin-Yue Qiao^1,2 †^, Jia-Wei Xiong^2, †^, Yi-Xiang Chen^*1,2,3^, Jan C. M. De Hoog^4^, Julian Pearce^5^, Fang Huang^1,2,3^, Zi-Fu Zhao^1,2^, Kun Chen^1,2^

^1^ State Key Laboratory of Lithospheric and Environmental Co-evolution, University of Science and Technology of China, Hefei 230026, China

^2^ School of Earth and Space Sciences, University of Science and Technology of China, Hefei 230026, China

^3^ Deep Space Exploration Laboratory, Hefei 230026, China

^4^ School of GeoSciences, Grant Institute, University of Edinburgh, James Hutton Road, EH9 3FE, United Kingdom

^5^ School of Earth and Ocean Sciences, Cardiff University, Main Building, Park Place, Cardiff CF10 3AT, United Kingdom

Corresponding author: Yi-Xiang Chen; Email: yxchen07@ustc.edu.cn.

^†^These authors contributed equally to this work.

**This PDF file includes:**

Methods for geochemical modelling details shown in Supplementary Figures S5-S7;

Supplementary Figures S1 to S8;

Supplementary Tables S1 to S3.

Supplementary Dataset in another file (Excel).

**Methods**

**Mélange melting model details for the trace element compositions**

To better constrain the contribution of sediment components to SSI arc magma formation, we conducted batch and fractional melting modelling of serpentinite-dominated mélange. Using the partition coefficients from Workman and Hart [1], we assumed the mélange predominantly comprises serpentinite with a minor sediment fraction ranging from 1% to 5% in mass proportion. The combined Mg-B isotopic constraint requires a composite source consisting of ~20 to 70% mélange composition to account for the observed compositions in SSI arc magmas (Fig. 4C–D). Thus, we assumed a composite source comprised of 50% mélange and 50% depleted mantle (DM) [1]. The initial trace element compositions for forearc serpentinite in this study are from Peace *et al.* [2], which are listed in the Supplementary Dataset. Trace element compositions for sediments in SSI setting are from Barry [3] and Plank [4]. The calculated trace element compositions are normalized to average values of the studied SSI arc magmatic rocks (MgO contents >5 wt%, n=9). The results show that a minor sediment contribution (<3% by mass proportion) adequately explains the majority of trace element compositions observed in SSI arc magmas (Fig. S5). Some incompatible elements like Pb and Hf is not shown due to the lack of data in SSI forearc serpentinite. Deviation in elements such as Sr, Rb and Ba may have resulted from the influence of forearc serpentinite, which experienced remarkable enrichment of these fluid-mobile elements during serpentiniztaion [5]. It is noteworthy that these fluid-mobile elements would be lost with the progressive dehydration of forearc serpentinite.

**Sr-Nd isotope mixing model**

We further evaluated the contribution of sediment components in the context of Sr-Nd isotope systematics (Fig. S6). **Details of the mantle and sediment end-member compositions can be found in the** Supplementary Dataset**.** Since Sr-Nd isotope data for SSI forearc serpentinite are unavailable, we alternatively utilized data from the global serpentinite as the endmember [1,6]. Similar to trace element modelling, we assumed a mélange primarily consisting of forearc serpentinite and sediment, and the SSI arc magmas sourced from a composite source comprises 50% mélange and 50% DM. The SSI sediment compositions are from Barry *et al.* [3] and the average compositions are used. Due to the strong fractionation of Sr and Nd elements during partial melting of sediments, the mixing between depleted mantle, bulk sediment, and sediment melts (showed dashed lines) show different trends. The partition coefficients for Sr and Nd during partial melting of sediments at 750-900°C are set as 7.3 and 0.35, respectively, according to Hermann and Rubatto [7]. The modelling results shown in Fig. S6 suggests that the consideration of serpentinite in the subarc mantle source is also consistent with the addition of sediment in the form of bulk solid rather than in the form of sediment melt.

**Phase equilibrium modelling for sediment melting**

To constrain the stability of rutile during sediment partial melting, we have conducted the P-T pseudosection phase equilibrium modelling for the sediment from the South Sandwich subduction zone [4]. The modelling is for the system NCKFMASHTO (Na_2_O-CaO-K_2_O-FeO-MgO-Al_2_O_3_-SiO_2_-H_2_O-O) using GeoPS 3.5 [8] with the internally consistent thermodynamic dataset ds62 [9]. The activity-composition models are used as follows: biotite, muscovite, paragonite, garnet and silicate melt [10], ilmenite [11], jadeite [12], glaucophane [13], plagioclase, feldspar [14], epidote [9]. Coesite, quartz, rutile, lawsonite and kyanite are treated as pure end-member phases. The bulk sediment compositions for calculations are from Plank [4] and summarized in Supplementary Dataset. Different Fe^3+^/(Fe^3+^ + Fe^2+^) ratios (0.05 and 0.20) are considered to constrain the effect of redox state variation. Considering that the sediment will experience water loss at subarc depths, a lower bulk water content than that reported by Plank [4] is used. Notably, the water content variations do not significantly affect the stability of rutile. The modelling results consistently indicate that rutile is always a stable phase during sediment partial melting, regardless of the Fe^3+^/(Fe^3+^ + Fe^2+^) ratios (Fig. S7). Rutile occurs at higher pressure under more oxidized conditions, which is consistent with previous study [10].


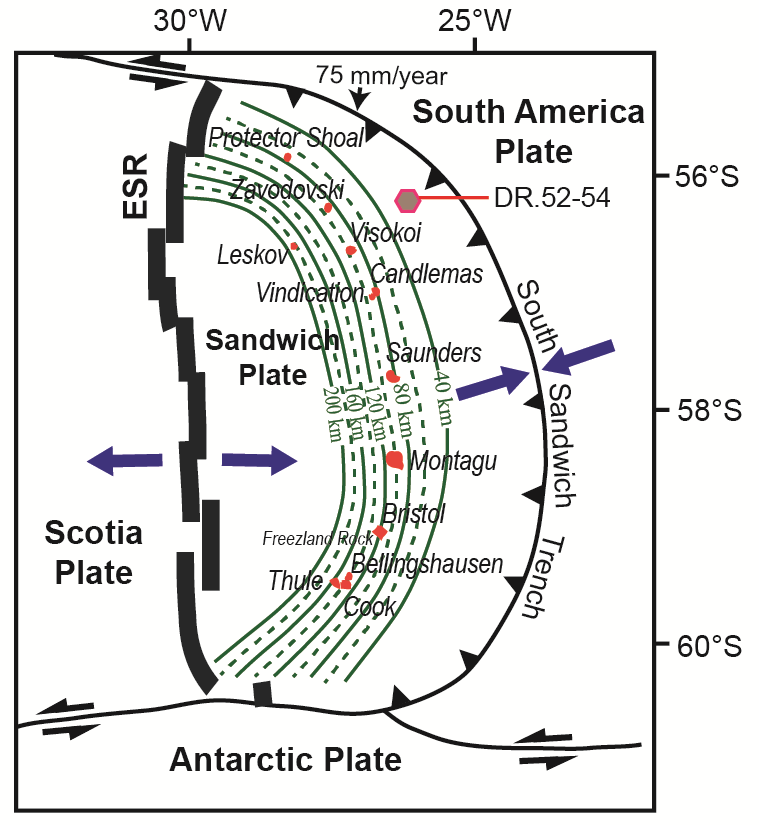


Supplementary Fig. S1. The contoured depths of the subducted South America slab beneath the South Sandwich Island arc, based on the results of Slab2 model [15].


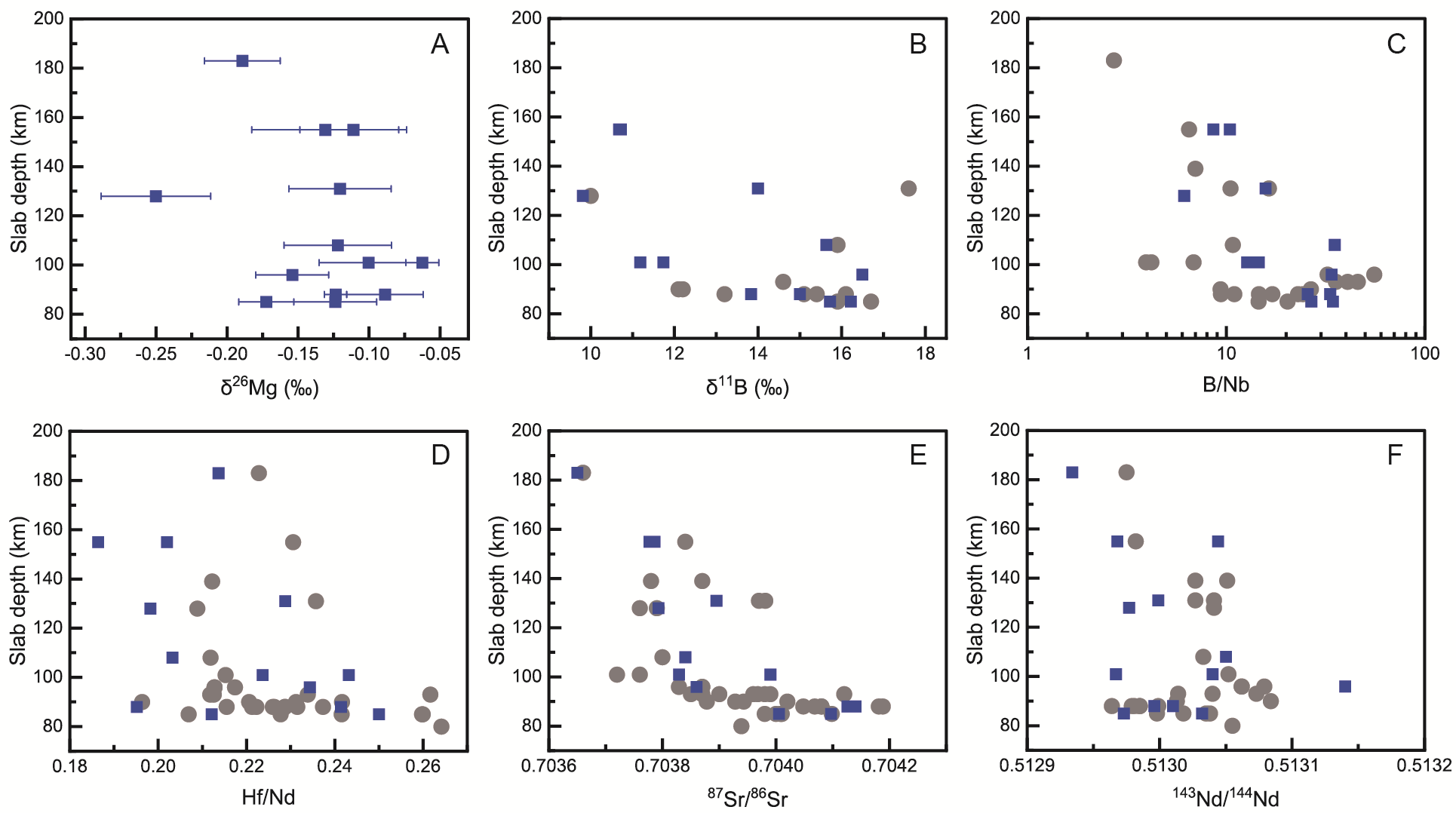


Supplementary Fig. S2. Slab depth versus δ^26^Mg (A), δ^11^B (B), B/Nb (C), Hf/Nd (D), ^87^Sr/^86^Sr (E)and ^143^Nd /^144^Nd (F) for the SSI arc volcanic rocks. The data in this study are in dark blue, and the literature data for SSI arc magmas (gray circles) are from Pearce *et al.* [16], Barry *et al*. [3] and Tonarini *et al.* [17]. Note the analytical uncertainty in δ^11^B are smaller than the symbols.


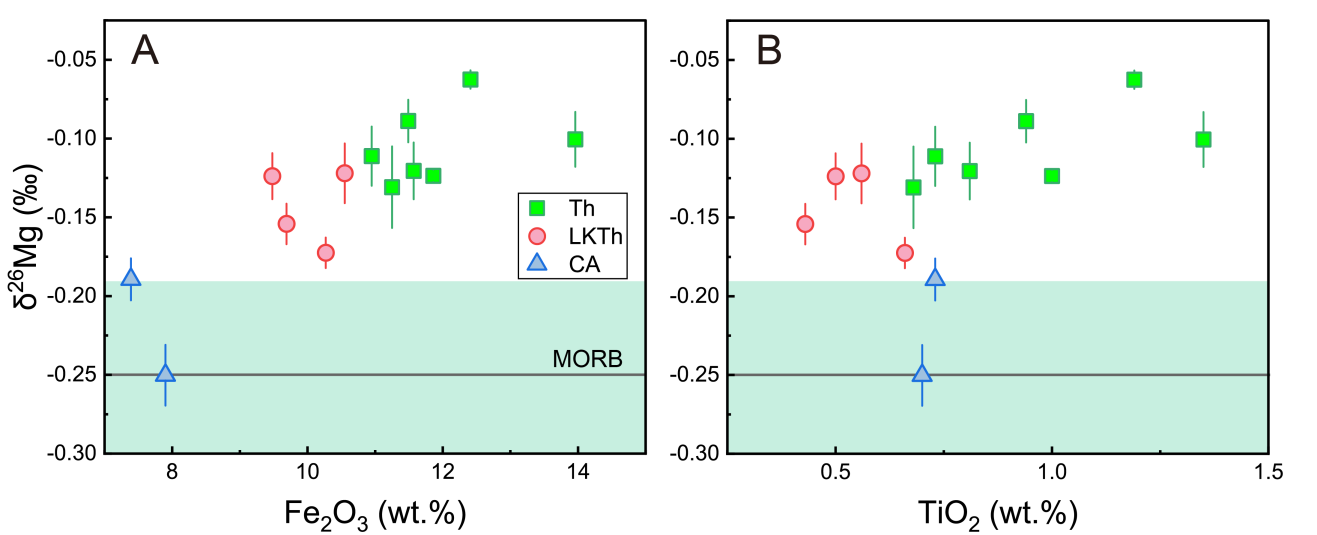


Supplementary Fig. S3. δ^26^Mg versus Fe_2_O_3_ (A) and TiO_2_ contents (B) for the SSI arc volcanic rocks. TH, tholeiite; LKTh, low-K tholeiite; CA, calc-alkaline magma.


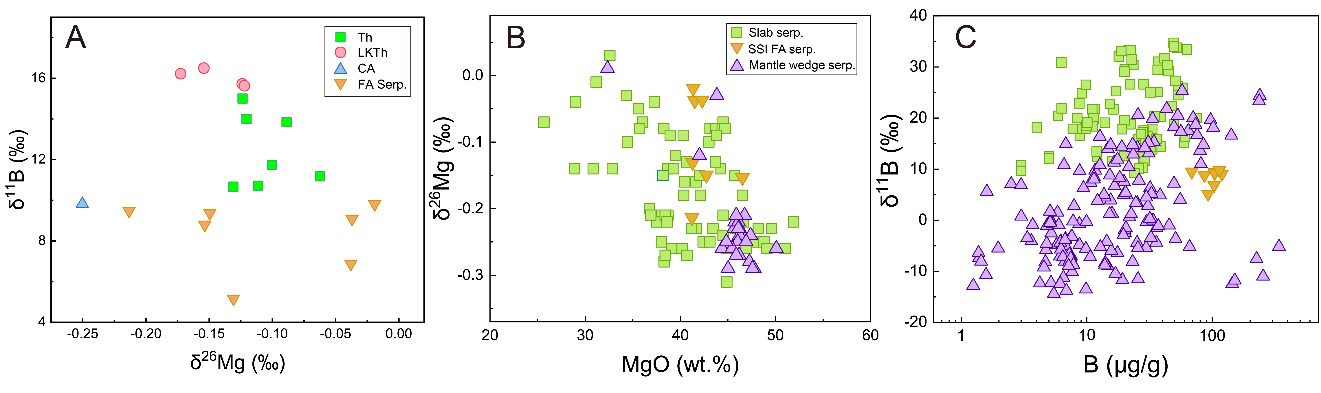


Supplementary Fig. S4. Magnesium and B elemental and isotopic compositions of the studied samples and of relevant serpentinites from the literature. (A) δ^11^B versus δ^26^Mg of the studied samples. TH, tholeiite; LKTh, low-K tholeiite; CA, calc-alkaline magma. (B) δ^26^Mg versus MgO contents of SSI forearc serpentinites and related serpentinites in literature [18-22]. (C) δ^11^B versus B contents of SSI forearc serpentinites and related serpentinites in literature [23-26]. Source data are provided in Supplementary Dataset.

**
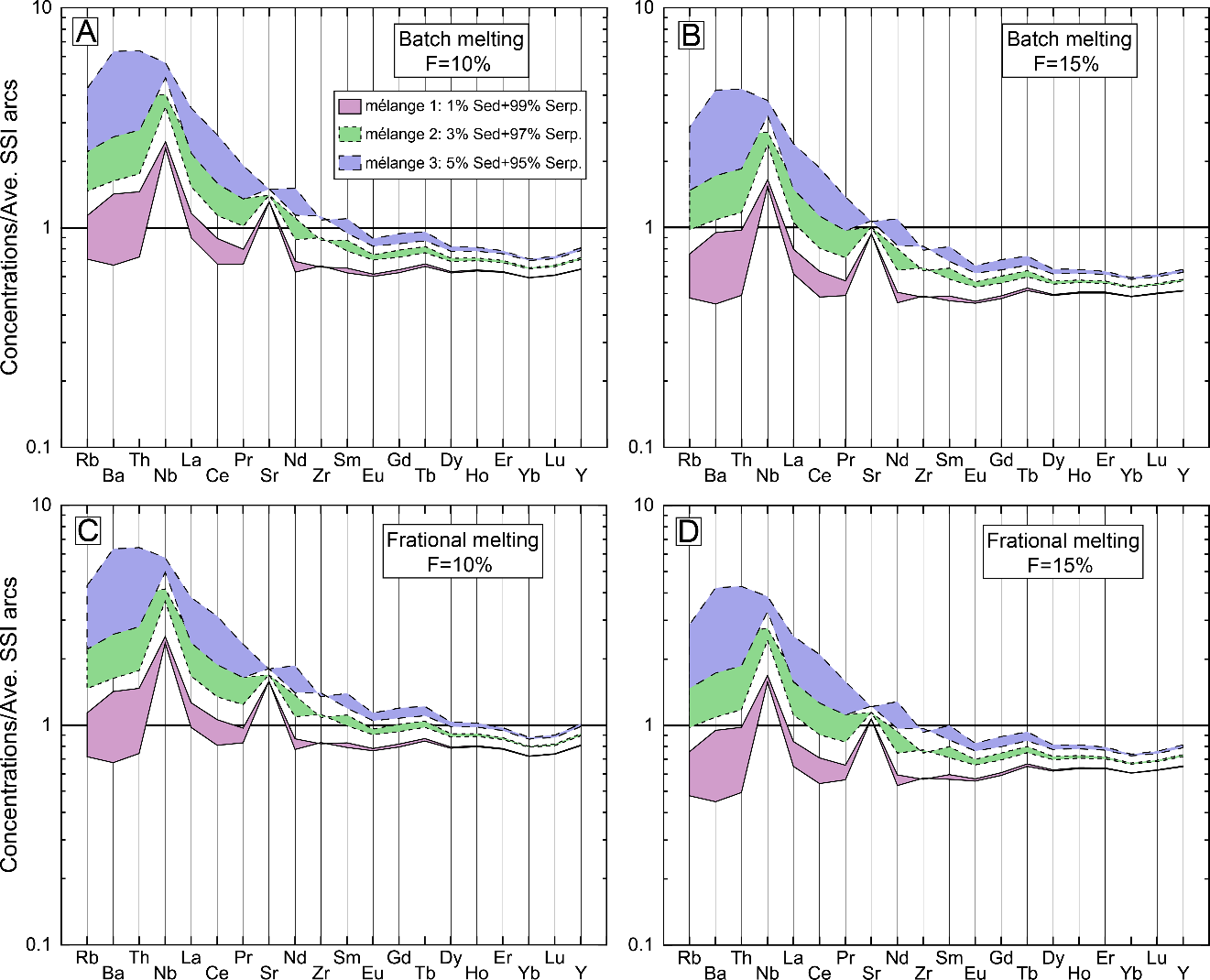
**

Supplementary Fig. S5. Trace element evidence for the mélange melting model. (A–B) Results of batch melting model with melting degree of 10% and 15%, respectively. (C–D) Results of fractional melting model with melting degree of 10% and 15%, respectively. Concentrations are normalized to the average high-MgO (> 5 wt%) SSI arc rocks in this study. The calculated melts are assumed to be derived from a composite source comprising 50% mélange and 50% depleted mantle rocks, as constrained by Mg-B isotopes results in Fig. 3. See methods section in Supporting Information for details.


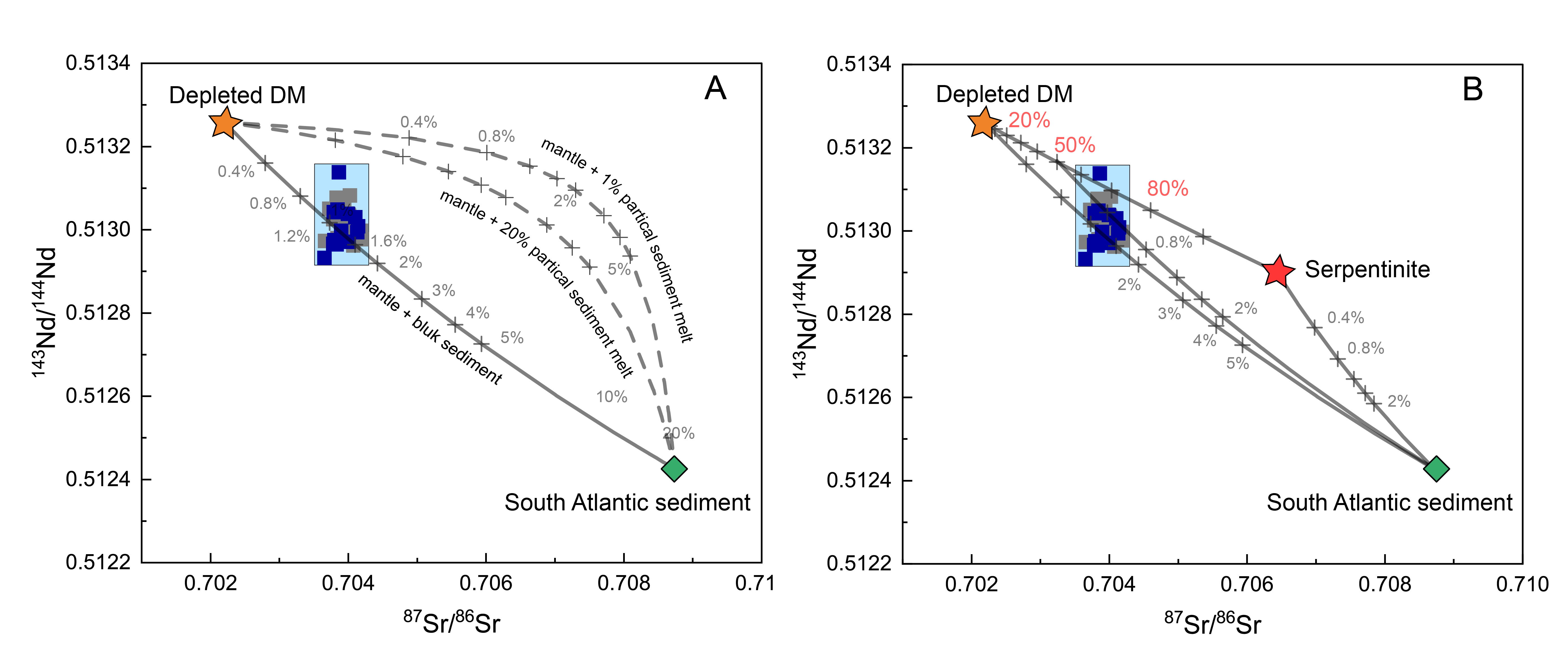


Supplementary Fig. S6. Binary mixing model for Sr-Nd isotopes of SSI arc rocks. The numbers along the mixing lines represent mass proportion of sediment. Data for forearc serpentinites are from Xiong *et al.* [6]. The sediment compositions associated with SSI arcs are averaged from Barry *et al.* [3]. The other data of SSI arc magmas (gray) are from Pearce *et al.* [15], Barry *et al*. [3] and Tonarini *et al.* [17]. See methods section in Supporting Information for details. For clarity, the mixing curves of depleted mantle with sediment melts are not shown in panel B. This figure suggests that the inclusion of serpentinite in the subarc mantle source (even when considering high ^87^Sr/^86^Sr ratios that may be induced by serpentinization) is also consistent with the addition of sediment in the form of bulk solid rather than as sediment melt.


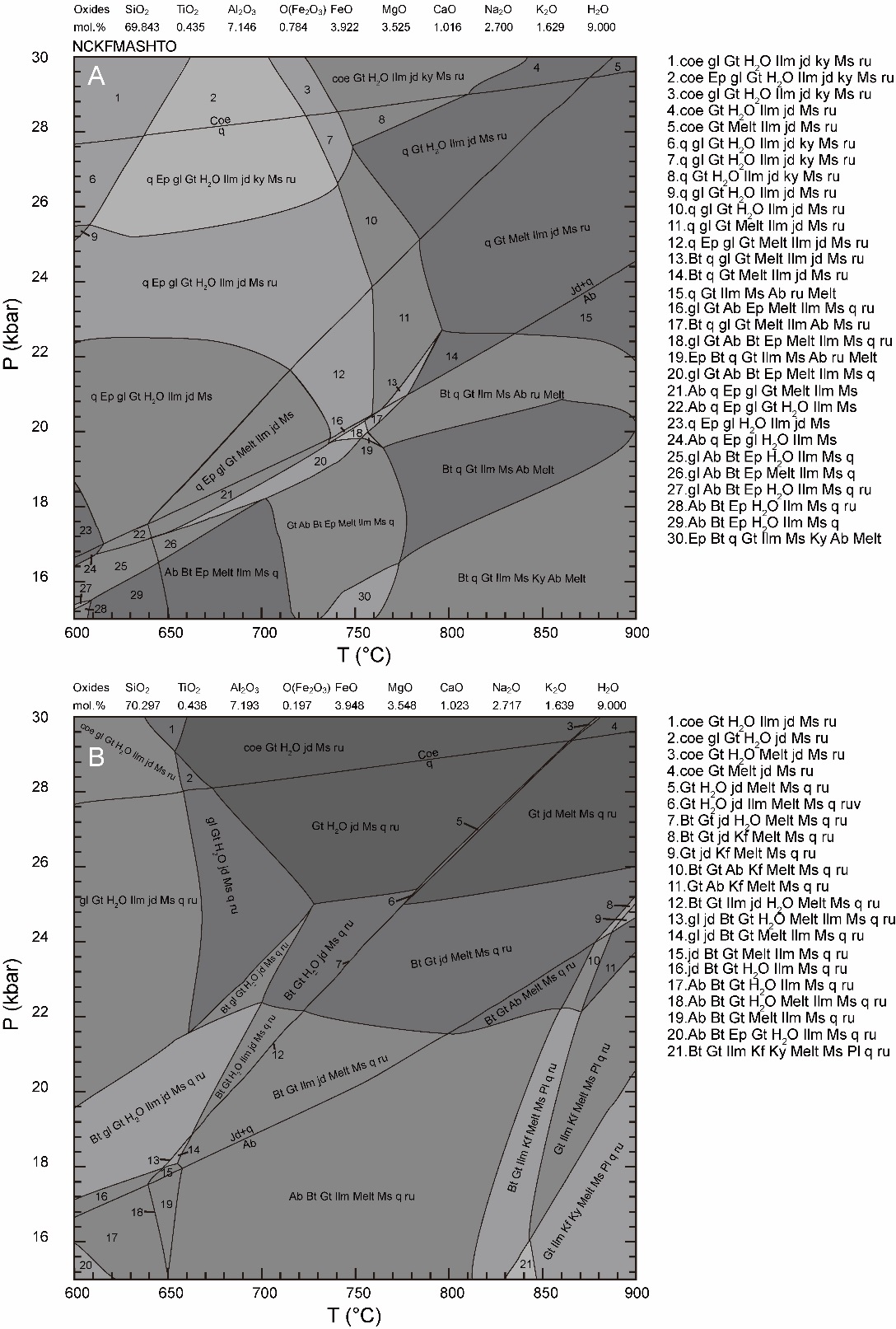


Supplementary Fig. S7. Pseudosection phase equilibrium modelling for South Sandwich sediments. See methods section in Supporting Information for modelling details. Mineral abbreviations: Ab, Albite; Bt, Biotite; Coe, Coesite; Ep, Epidote; Gl, Glaucophane; Gt, Garnet; Ilm, Ilmenite; Jd, Jadeite; Kf, K-feldspar; Ky, Kyanite; Ms, Muscovite; Pl, Plagioclase; q, Quartz; ru, Rutile.


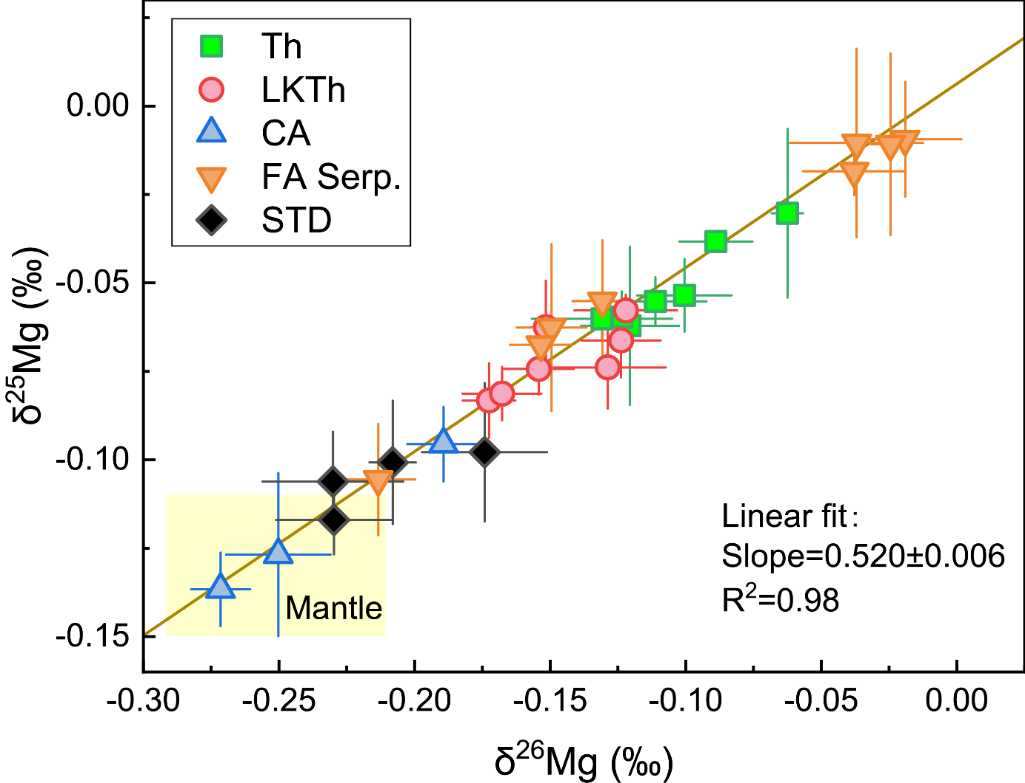


Supplementary Fig. S8. Diagram of δ^25^Mg versus δ^26^Mg for the SSI arc volcanic rocks, forearc serpentinites (FA Serp.) and rock standards (STD). TH, tholeiite; LKTh, low-K tholeiite; CA, calc-alkaline magma.

Supplementary Table S1. Whole-rock boron isotope compositions of arc rocks and forearc serpentinites from the SSI arc system

| **Samples** | **Rock type** | **B (ppm)** | **δ^11^B (‰)** | **2SE (‰)** |
| --- | --- | --- | --- | --- |
| **SSI arc lavas** | | | | |
| SSC34.2 | LKTh | 7.22 | +16.2 | 0.06 |
| SSC35.4 | LKTh | 5.09 | +15.7 | 0.08 |
| SSM1.3 | LKTh | 7.12 | +16.5 | 0.06 |
| SSR1.3 | LKTh | 8.07 | +15.6 | 0.08 |
| SSS5.4 | Th | 18.7 | +13.8 | 0.10 |
| SSS15.1 | Th | 17.7 | +15.0 | 0.08 |
| SSB12.1 | Th | 13.6 | +14.0 | 0.08 |
| SST6.3 | Th | 6.88 | +10.7 | 0.10 |
| SST7.1 | Th | 7.84 | +10.7 | 0.08 |
| SSW6.3 | Th | 13.9 | +11.7 | 0.10 |
| SSW9.3 | Th | 14.0 | +11.2 | 0.08 |
| replicate |  | 14.3 | +11.3 | 0.10 |
| SSF1.3 | CA | 11.7 | +9.81 | 0.06 |
| replicate |  | 10.9 | +9.58 | 0.08 |
| **Dredged forearc serpentinites** | | | | |
| DR.52.11 | Hz | 68.6 | +9.49 | 0.08 |
| DR.52.12 | Hz | 105.4 | +9.38 | 0.10 |
| replicate |  | 104.0 | +9.30 | 0.10 |
| DR.54.10 | Trans. | 92.6 | +5.16 | 0.10 |
| DR.52.4 | Trans. | 103.5 | +6.88 | 0.08 |
| DR.52.5 | Dunite | 86.7 | +8.80 | 0.08 |
| DR.53.2 | Dunite | 113.8 | +9.82 | 0.10 |
| DR.53.3 | Dunite | 119.4 | +9.09 | 0.10 |
| **Reference materials** | |  |  |  |
| BHVO-2 |  | 2.71 | -1.30 | 0.08 |
| B5 |  | 9.06 | -4.07 | 0.10 |
| JA-2 |  | 21.6 | -8.55 | 0.08 |
| JB-2 |  | 29.0 | +6.30 | 0.08 |
| JR-2 |  | 151.1 | +3.07 | 0.08 |

Abbreviation: LKTh, low-K tholeiitic basalt; Th, Tholeiitic basalt; CA, Calc-alkaline magma; Hz, harzburgite; Trans, transitional peridotite.

Supplementary Table S2. Whole-rock Mg, Sr and Nd isotope compositions of the SSI arc volcanic rocks and forearc serpentinites

| Sample | Rock type | MgO (wt%) | δ^26^Mg (‰) | 2SD (‰) | δ^25^Mg (‰) | 2SD (‰) | N | ^87^Sr/^86^Sr | ^143^Nd/^144^Nd | Ref. |
| --- | --- | --- | --- | --- | --- | --- | --- | --- | --- | --- |
| **SSI arc lavas** | | | | | | | | | | |
| SSC34.2 | LKTh | 6.27 | -0.17 | 0.02 | -0.08 | 0.02 | 3 | 0.70401* | 0.51297* | 3 |
| SSC35.4 | LKTh | 6.34 | -0.12 | 0.03 | -0.07 | 0.02 | 3 | 0.70410* | 0.51303* | 16 |
| replicate |  |  | -0.15 | 0.01 | -0.06 | 0.03 | 3 |  |  |  |
| SSM1.3 | LKTh | 10.8 | -0.15 | 0.03 | -0.07 | 0.01 | 3 |  |  |  |
| replicate |  |  | -0.17 | 0.03 | -0.08 | 0.02 | 3 | 0.70386* | 0.51314* | 3 |
| replicate |  |  | -0.13 | 0.04 | -0.07 | 0.02 | 3 |  |  |  |
| SSR1.3 | LKTh | 5.89 | -0.12 | 0.04 | -0.06 | 0.01 | 3 | 0.70384* | 0.51305* | 3 |
| SSB12.1 | Th | 3.89 | -0.12 | 0.04 | -0.06 | 0.04 | 3 | 0.70390* | 0.51300* | 16 |
| SSS5.4 | Th | 5.51 | -0.09 | 0.03 | -0.04 | 0.00 | 3 | 0.70414* | 0.51301* | 3 |
| SSS15.1 | Th | 6.02 | -0.12 | 0.01 | -0.06 | 0.01 | 3 | 0.70413 | 0.51300 |  |
| SST6.3 | Th | 6.19 | -0.13 | 0.05 | -0.06 | 0.01 | 3 | 0.70378* | 0.51297* | 16 |
| SST7.1 | Th | 5.11 | -0.11 | 0.04 | -0.06 | 0.01 | 3 | 0.70379* | 0.51304* | 3 |
| SSW6.3 | Th | 4.19 | -0.10 | 0.03 | -0.05 | 0.02 | 3 | 0.70399* | 0.51304* | 3 |
| SSW9.3 | Th | 5.32 | -0.06 | 0.01 | -0.03 | 0.05 | 3 | 0.70383* | 0.51297* | 16 |
| SSF1.3 | CA | 2.61 | -0.25 | 0.04 | -0.13 | 0.05 | 3 | 0.70379* | 0.51298* | 16 |
| replicate |  |  | -0.27 | 0.02 | -0.14 | 0.02 | 3 |  |  |  |
| SSL12.1 | CA | 3.82 | -0.19 | 0.03 | -0.10 | 0.02 | 3 | 0.70365 | 0.51293 |  |
| **Dredged forearc peridotites** | | | | | | | | | | |
| DR.52.11 | Hz | 41.2 | -0.21 | 0.03 | -0.11 | 0.03 | 3 |  |  |  |
| DR.52.12 | Hz | 42.7 | -0.15 | 0.03 | -0.06 | 0.05 | 3 |  |  |  |
| DR.52.4 | Trans. | 41.5 | -0.04 | 0.04 | -0.02 | 0.01 | 3 |  |  |  |
| DR.54.10 | Trans. | 41.3 | -0.13 | 0.02 | -0.06 | 0.03 | 3 |  |  |  |
| DR.53.3 | Dunite | 42.3 | -0.04 | 0.05 | -0.01 | 0.05 | 3 |  |  |  |
| DR.52.5 | Dunite | 46.5 | -0.15 | 0.02 | -0.07 | 0.02 | 3 |  |  |  |
| DR.53.2 | Dunite | 41.4 | -0.02 | 0.04 | -0.01 | 0.03 | 3 |  |  |  |
| replicate |  |  | -0.02 | 0.01 | -0.01 | 0.05 | 3 |  |  |  |
| **Reference materials** | | | | | | | | | | |
| BCR-2 | Basalt | 3.60 | -0.21 | 0.02 | -0.10 | 0.03 | 3 | 0.70502 | 0.51265 |  |
| BHVO-2 | Basalt | 7.26 | -0.23 | 0.05 | -0.11 | 0.03 | 3 | 0.70348 | 0.51299 |  |
| replicate |  |  | -0.24 | 0.01 | -0.13 | 0.01 | 3 |  |  |  |
| replicate |  |  | -0.23 | 0.04 | -0.12 | 0.02 | 3 |  |  |  |
| AGV-2 | Andesite | 1.80 | -0.17 | 0.05 | -0.10 | 0.04 | 3 |  |  |  |

Note: *Data from the mentioned references. Abbreviation: LKTh, low-K tholeiitic basalt; Th, Tholeiitic basalt; CA, Calc-alkaline magma; Hz, harzburgite; Trans, transitional peridotite.

Supplementary Table S3. Parameters for the mixing models in Fig. 3

| Endmember  composition | B | δ^11^B | Nb | Nb/B | MgO | δ^26^Mg | Ref. |
| --- | --- | --- | --- | --- | --- | --- | --- |
|  | μg/g | ‰ | μg/g |  | wt% | ‰ |  |
| **Depleted MORB mantle** | | | | | | | |
| Initial composition | 0.077 | -7.1 | 0.148 | 1.9 | 38.7 | -0.25 | 1,16,21 |
| **Forearc Serpentinite** | | | | | | | |
| Initial composition | 60 | +22.0 |  |  | 40.4 | +0.03 |  |
| Final composition | 15 | +13.0 | 0.01 | 0.0007 | 40.4 | +0.03 | 25-33 |
| **Serpentinite-derived fluid** | | | | | | | |
| Fluid 1 |  |  |  |  | 1.05 | +0.15 |  |
| Fluid 2 |  |  |  |  | 4.35 | +0.15 | 34,35 |
| Fluid 3 |  |  |  |  | 5.00 | +0.50 |  |
| Fluid 4 | 325 | +19.0 | 0.33 | 0.001 |  |  |  |
| Fluid 5 | 306 | +15.0 | 0.31 | 0.001 |  |  | 17,36 |
| Fluid 6 | 289 | +10.8 | 0.29 | 0.001 |  |  |  |
| **Uppermost slab = 90%AOC+10%Sed** | | | | | | | |
| Initial composition |  |  |  |  |  |  |  |
| Sediment | 115 | -4.1 | 4.56 | 0.04 | 3.2 | +0.18 | 37,38 |
| AOC | 18 | +5.5 | 2.2 | 0.12 | 10.2 | +0.00 | 37-39 |
| Bulk slab | 28 | +2.4 | 2.4 | 0.09 | 9.5 | +0.10 |  |
| Slab-derived fluids | 170 | -3.9 | 1.7 | 0.01 | 0.5 | +0.20 | 17 |
| Residual slab | 16.8 | -12.8 | 1.68 | 0.1 | 9.5 | +0.10 | 17 |
| **Serpentinite-dominated mélange** | | | | | | | |
| 99% Serpentinite+1% Sediment | | | | | | | |
| Final composition | 16.6 | 10.6 | 1.2 | 0.071 | 38.5 | +0.031 |  |
| 95% Serpentinite+5% Sediment | | | | | | | |
| Final composition | 15.3 | 12.5 | 1.0 | 0.068 | 40.0 | +0.030 |  |

**References in Supplementary Information**

1. Workman R K and Hart S R. Major and trace element composition of the depleted MORB mantle (DMM). *Earth Planet Sci Lett* 2005; **231**: 53-72.
2. Pearce J A, Barker P F and Edwards S J *et al.* Geochemistry and tectonic significance of peridotites from the South Sandwich arc-basin system, South Atlantic. Contrib. *Mineral Petrol* 2000; **139**: 36-53.
3. Barry T L, Pearce J A and Leat P T *et al*. Hf isotope evidence for selective mobility of high-field-strength elements in a subduction setting: South Sandwich Islands. *Earth Planet Sci Lett* 2006; **252**: 223-44.
4. Plank T. The Chemical Composition of Subducting Sediments, *Treatise on Geochemistry (Second Edition)* 2014; 607-29.
5. Deschamps F, Godard M and Guillot S *et al*. Geochemistry of subduction zone serpentinites: A review. *Lithos* 2013; ***178***: 96-127.
6. Xiong J-W, Chen Y-X and Scambelluri M *et al.* Fluid-metasomatized rocks with extremely low δ^26^Mg values in subducted oceanic lithosphere: Implications for mantle Mg isotope heterogeneity and the origin of low-δ^26^Mg magmas. *Geochim Cosmochim Acta* 2024; **371**: 111-125.
7. Hermann J and Rubatto D. Accessory phase control on the trace element signature of sediment melts in subduction zones. *Chem Geol* 2009; **265**: 512-26.
8. Xiang H and Connolly J A D. GeoPS: An interactive visual computing tool for thermodynamic modelling of phase equilibria. *J Metamorph Geol* 2022; **40**: 243-55.
9. Holland T J B and Powell R. An improved and extended internally consistent thermodynamic dataset for phases of petrological interest, involving a new equation of state for solids. *J Metamorph Geol* 2011; **29**: 333-83.
10. White R W, Powell R and Holland T J B *et al.* New mineral activity-composition relations for thermodynamic calculations in metapelitic systems. *J Metamorph Geol* 2014; **32**: 261-86.
11. White R W, Powell R and Holland T J B *et al.* The effect of TiO_2_ and Fe_2_O_3_ on metapelitic assemblages at greenschist and amphibolite facies conditions:: mineral equilibria calculations in the system K_2_O-FeO-MgO-Al_2_O_3_-SiO_2_-H_2_O-TiO_2_-Fe_2_O_3_. *J Metamorph Geol* 2000; **18**: 497-511.
12. Green E, Holland T and Powell R. An order-disorder model for omphacitic pyroxenes in the system jadeite-diopside-hedenbergite-acmite, with applications to eclogitic rocks. *Am Mineral* 2007; **92**: 1181-9.
13. Green E C R, White R W and Diener J F A *et al.* Activity-composition relations for the calculation of partial melting equilibria in metabasic rocks. *J Metamorph Geol* 2016; **34**: 845-69.
14. Holland T and Powell R. Activity-composition relations for phases in petrological calculations: an asymmetric multicomponent formulation. *Contrib Mineral Petrol* 2003; **145**: 492-501.
15. Hayes G P, Moore G L and Portner D E et al. Slab2, a comprehensive subduction zone geometry model. Science 2018; 362: 58-61.
16. Pearce J A, Baker P E and Harvey P K *et al*. Geochemical evidence for subduction fluxes, mantle melting and fractional crystallization beneath the South Sandwich-Island Arc. *J Petrol* 1995; **36**: 1073-109.
17. Tonarini S, Leeman W P and Leat P T. Subduction erosion of forearc mantle wedge implicated in the genesis of the South Sandwich Island (SSI) arc: Evidence from boron isotope systematics. *Earth Planet Sci Lett* 2011; **301**: 275-84.
18. Eom J, Yoshimura T and Akizawa N *et al*. The magnesium isotopic compositions of the crust and mantle: A study on the Oman ophiolite. *Chem Geol* 2022; **606**: 120969.
19. Wang Y, Deng J and Liao R *et al**.* Magnesium isotopic composition of the Mariana forearc serpentinite: Implications for Mg isotopic composition of the mantle wedge and Mg isotopic fractionation during mantle wedge serpentinization. *Chem Geol* 2023; **624**: 121428.
20. Liu P-P, Teng F-Z and Dick H J B *et al*. Magnesium isotopic composition of the oceanic mantle and oceanic Mg cycling. *Geochim Cosmochim Acta* 2017; **206**: 151-65.
21. Teng F-Z, Li W-Y and Ke S *et al.* Magnesium isotopic composition of the Earth and chondrites. *Geochim Cosmochim Acta* 2010; **74**: 4150-66.
22. Li X, Li S and Zhang Z *et al.* Magnesium isotopic fractionation during post-serpentinization alteration: Implications for arc and oceanic Mg cycles. *Chem Geol* 2024; **648**: 121866.
23. Harvey J, Savov I P and Agostini S *et al*. Si-metasomatism in serpentinized peridotite: The effects of talc-alteration on strontium and boron isotopes in abyssal serpentinites from Hole 1268a, ODP Leg 209. *Geochim Cosmochim Acta* 2014; **126**: 30-48.
24. Yamada C, Tsujimori T and Chang Q *et al*. Boron isotope variations of Franciscan serpentinites, northern California. *Lithos* 2019; **334-5**: 180-9 (2019).
25. Scambelluri M and Tonarini S. Boron isotope evidence for shallow fluid transfer across subduction zones by serpentinized mantle. *Geology* 2014; **40**: 907-10.
26. Benton L D, Ryan J G and Tera F. Boron isotope systematics of slab fluids as inferred from a serpentine seamount, Mariana forearc. *Earth Planet Sci Lett* 2001; **187**: 273-82.
27. Savov I P, Ryan J G and D'Antonio M *et al*. Shallow slab fluid release across and along the Mariana arc-basin system: Insights from geochemistry of serpentinized peridotites from the Mariana fore arc. *J Geophys Res* 2007; **112**: https://doi.org/10.1029/2006JB004749.
28. Scambelluri M, Pettke T and Cannao E. Fluid-related inclusions in Alpine high-pressure peridotite reveal trace element recycling during subduction-zone dehydration of serpentinized mantle (Cima di Gagnone, Swiss Alps). *Earth Planet Sci Lett* 2015; **429**: 45-59.
29. Vils F, Tonarini S and Kalt A et al. Boron, lithium and strontium isotopes as tracers of seawater-serpentinite interaction at Mid-Atlantic ridge, ODP Leg 209. *Earth Planet Sci Lett* 2009; **286**: 414-25.
30. Kodolányi J and Pettke T. Loss of trace elements from serpentinites during fluid-assisted transformation of chrysotile to antigorite — An example from Guatemala. *Chem Geol* 2011; **284**: 351-62.
31. Scambelluri M, Bottazzi P and Trommsdorff V *et al*. Incompatible element-rich fluids released by antigorite breakdown in deeply subducted mantle. *Earth Planet Sci Lett* 2001; **192**: 457-70.
32. Boschi C, Bonatti E and Ligi M *et al*, Serpentinization of mantle peridotites along an uplifted lithospheric section, Mid Atlantic Ridge at 11° N. *Lithos* 2013; **178**: 3-23.
33. Boschi C, Dini A and Früh-Green G L *et al*. Isotopic and element exchange during serpentinization and metasomatism at the Atlantis Massif (MAR 30°N): Insights from B and Sr isotope data. *Geochim Cosmochim Acta* 2008; **72**: 1801-23.
34. Jones R E, De Hoog J C M and Kirstein L A *et al.* Temporal variations in the influence of the subducting slab on Central Andean arc magmas: Evidence from boron isotope systematics. *Earth Planet. Sci. Lett.* 2014; **408**: 390-401.
35. Chen Y-X, Schertl H-P and Zheng Y-F *et al.* Mg-O isotopes trace the origin of Mg-rich fluids in the deeply subducted continental crust of Western Alps. *Earth Planet Sci Lett* 2016; **456**: 157-67.
36. Kessel R, Schmidt M W and Ulmer P *et al.* Trace element signature of subduction-zone fluids, melts and supercritical liquids at 120-180 km depth. *Nature* 2005; **437**: 724-7.
37. Hu Y, Teng F-Z and Plank T *et al.* Magnesium isotopic composition of subducting marine sediments. *Chem Geol* 2017; **466**: 15-31.
38. Huang K-J, Teng F-Z and Plank T *et al.* Magnesium isotopic composition of altered oceanic crust and the global Mg cycle. *Geochim Cosmochim Acta* 2018; **238**: 357-73.
39. Liao R, Zhu H and Zhang L *et al.* Unusual δ^26^Mg values in oceanic crust basalts from the South China Sea. *GSA Bulletin* 2022; **135**: 523-33.
